# Morphologic changes in the visual cortex of patients with anisometropic amblyopia: a surface-based morphometry study

**DOI:** 10.1186/s12868-019-0524-6

**Published:** 2019-08-02

**Authors:** Minglong Liang, He Xiao, Bing Xie, Xuntao Yin, Jian Wang, Hong Yang

**Affiliations:** 1 Department of Radiology, Southwest Hospital, Army Medical University, Chongqing, China; 2 Department of Radiology, Aviation Medical Evaluation & Training Center of Airforce in Hangzhou, Hangzhou, Zhejiang China; 3 Department of Outpatient, Southwest Hospital, Army Medical University, Chongqing, China; 4 Department of Ophthalmology, Daping Hospital, Army Medical University, Chongqing, China

**Keywords:** Anisometropic amblyopia, Visual cortex, SBM, FreeSurfer, Cortical thickness, Mean curvature

## Abstract

**Background:**

Amblyopia is generally considered a neurodevelopmental disorder that results from abnormal visual experiences in early childhood and may persist to adulthood. The neural basis of amblyopia has been a matter of interest for many decades, but the critical neural processing sites in amblyopia are not entirely understood. Although many functional neuroimaging studies have found abnormal neuronal responses both within and beyond V1, few studies have focused on the neurophysiologic abnormalities in the visual cortex from the viewpoint of potential structural reorganization. In this study, we used a well-validated and highly accurate surface-based method to examine cortical morphologic changes in the visual cortex using multiple parameters (including cortical thickness, surface area, volume and mean curvature).

**Results:**

The cortical thicknesses of the bilateral V1, left V2, left ventral V3, left V4 and left V5/MT+ in patients were significantly thinner than that in controls. The mean curvature of the bilateral V1 was significantly increased in the patients compared with the controls. For the surface area and gray matter volume, no significant differences were found between patients and controls in all region of interests. The cortical thicknesses of the bilateral V1 were both negatively correlated with the amount of anisometropia. No significant correlations were found between any other surface parameters and clinical variables.

**Conclusion:**

In addition to cortical thickness, the altered mean curvature of the cortex may indicate neuroanatomic impairments of the visual cortex in patients with anisometropic amblyopia. Moreover, the structural changes were bilateral in the primary visual cortex but were unilateral in the secondary and more senior visual cortex.

## Background

Amblyopia is a developmental disorder of vision that is believed to follow from abnormal binocular interactions or visual deprivation during early life [1]. It is typically divided into different categories based on the eye disorder responsible for disrupting visual development, and anisometropic amblyopia is one of the most common types [2]. However, amblyopia is attributed to neurological abnormalities in the brain rather than abnormalities in the eye [1].

In the past few decades, significant abnormalities have been found that promoted our understanding of the neural mechanisms of amblyopia, but the critical neural processing sites in amblyopia remain unclear. A number of studies have shown no significant anatomic or physiologic deficits in the retina [3]. Extensive neuroimaging studies have found the loci and extent of cortical dysfunctions in amblyopic patients using techniques such as positron emission tomography, functional magnetic resonance imaging (fMRI) and magnetoencephalography with a variety of visual stimuli [4]. Most recently, several resting-state fMRI studies have found altered functional connectivity and spontaneous brain activity patterns in the visual areas in individuals with anisometropic amblyopia [5, 6].

Although many functional neuroimaging studies have found abnormal neuronal responses both within and beyond V1, only a few studies have focused on the neurophysiologic abnormalities of the visual cortex from the viewpoint of potential structural reorganization. Two studies with the voxel-based morphometry (VBM) technique have indicated that adults and children with amblyopia have reduced gray matter volume in visual cortical regions [7, 8]. Subsequently, through the cortical thickness approach, a comparison between amblyopic patients and normal controls found cortical thinning of occipital lobe in anisometropic amblyopic children [9]. However, these results were based on a rough anatomic atlas so that they cannot reflect accurate structural alterations in each visual area. Meanwhile, all these studies were confined to imprecise methods (i.e., VBM approach) [10] or a unitary parameter (i.e., gray matter volume or cortical thickness), which may have reduced the persuasiveness of their findings. Moreover, none of these studies examined the relationship between brain structural alterations and clinical measurements.

In the present study, a well-validated and highly accurate surface-based method [11] was therefore used to examine cortical morphologic changes in the visual cortex using multiple parameters (including cortical thickness, surface area, volume and mean curvature). Additionally, the correlations between the morphologic parameters in these visual areas and clinical measurements were also investigated.

## Results

### Demographic and clinical variables

The demographic characteristics and clinical variables are presented in Table 1. No significant differences were found in age, sex, education, laterality of the amblyopic/nondominant eye between the patients and controls. In the patients, the corrected visual acuity (cVA) of the amblyopic eye was significantly lower than that of the fellow eye, whereas there was no significant difference in cVA between the dominant and nondominant eyes in the controls. In addition, the patients showed significantly more amount of anisometropia than controls. Moreover, no significant difference was found in retinal nerve fiber layer (RNFL) thickness between the amblyopic and fellow eyes in the patients.

**Table 1:** Demographic and clinical variables of the participants

Characteristics	Patients (n = 20)	Controls (n = 20)	*P* value
Age (years)	25.20 ± 9.94	26.70 ± 7.40	0.591^a^
Gender (male/female)	9/11	8/12	0.749^b^
Education (years)	12.95 ± 3.03	13.80 ± 2.38	0.330^a^
Amblyopia/nondominant eye of control (left/right)	12/8	16/4	0.168^b^
cVA (LogMAR)			
Amblyopic eye (nondominant eye of control)	0.70 (0.70)	0 (0.08)	< *0.001*^c^
Fellow eye (dominant eye of control)	0 (0.08)	0 (0.08)	0.975^c^
*P* value	< *0.001*^d^	> 0.999^d^	–
Amount of anisometropia (diopter)	2.75 (1.88)	0.13 (0.94)	< *0.001*^c^
RNFL thickness (μm)			
Amblyopic eye	102.80 ± 12.15	–	–
Fellow eye	105.00 ± 12.63	–	–
*P* value	0.268^e^	–	–

The measurement data are expressed as the mean ± standard deviation and median (inter-quartile range) for the normality and nonnormality data, respectively. The enumeration data are expressed as frequency number

*cVA* corrected visual acuity, *LogMAR* logarithm of the minimum angle of resolution, *RNFL* retinal nerve fiber layer

^a^The *P* value was calculated using independent-samples t tests

^b^The *P* value was calculated using the Chi-square test

^c^The *P* value was calculated using Mann–Whitney U test

^d^The *P* value was calculated using Wilcoxon’s signed rank test

^e^The *P* value was calculated using paired-samples t test

### Surface parameters

**Fig. 1:**
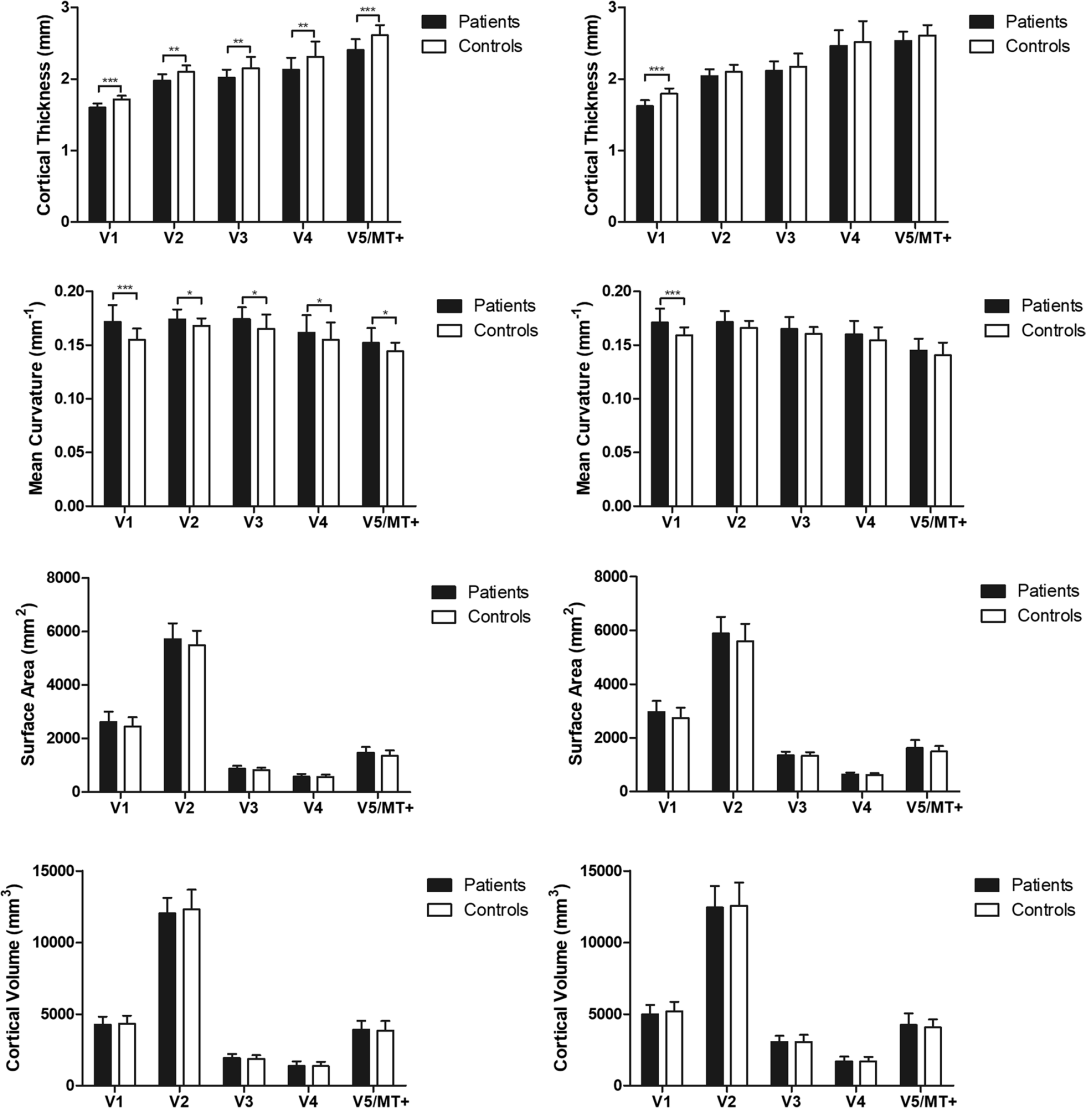
The differences in each surface parameter (cortical thickness, surface area, cortical volume and mean curvature) of each ROI between the patients and controls (*P < 0.05, **P < 0.01, ***P < 0.001)

The differences in each surface parameter (cortical thickness, surface area, cortical volume and mean curvature) of each region of interest (ROI) between the patients and controls are shown in Fig. 1. The patients showed significantly thinner cortical thickness than the controls in the bilateral V1, left V2, left ventral V3, left V4 and left V5/MT+ (*P *< 0.05/5, Bonferroni correction). The mean curvature of the bilateral V1 were significantly increased in patients compared with the controls (*P *< 0.05/5, Bonferroni correction). Although the mean curvature of the left V2, left ventral V3, left V4 and left V5/MT+ seemed to increase in the patients compared with the controls (*P *< 0.05), the results did not survive after Bonferroni correction (*P *> 0.05/5). For the other surface parameters, such as the surface area and gray matter volume, no significant differences were found between patients and controls in all ROIs.

### Correlation

**Fig. 2:**
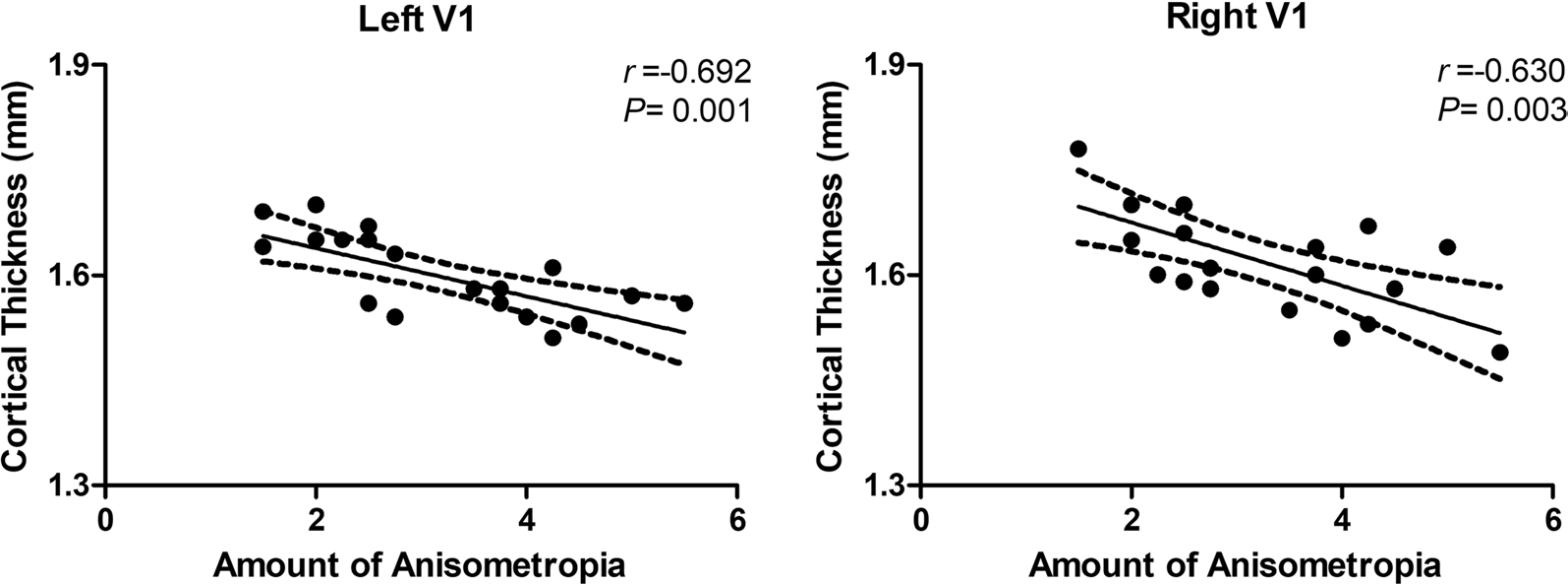
The correlation between the cortical thicknesses of the bilateral V1 and the amount of anisometropia quantified by the binocular difference in spherical equivalents

The cortical thicknesses of the bilateral V1 were both negatively correlated with the amount of anisometropia in the patients (Fig. 2). No significant correlations were found between any other surface parameters and any of the clinical variables.

## Discussion

In the present study, we analyzed the morphological alterations in the visual cortex in patients with anisometropic amblyopia using a structural MRI technique combined with the SBM approach. We compared the cortical thickness, surface area, volume and mean curvature of the visual cortex between amblyopic patients and normal-sight controls. The significantly thinner thickness of several visual areas in patients was generally consistent with the results of previous studies. The mean curvature of the same visual areas seemed to increase in the patients compared with the controls, while none but the mean curvature alterations in the bilateral V1 showed statistical significance. In addition, the cortical thicknesses of the bilateral V1 were negatively correlated with the amount of anisometropia.

The human visual cortex can be divided into several functional visual areas (such as V1, V2, V3) based on its inherent retinotopic organization [12]. It is generally agreed that the earliest functional and structural abnormalities that contribute significantly to the behavioral losses in amblyopia occur in V1. The pioneering work of Wiesel [13] and extensive subsequent work have demonstrated that abnormal visual experience results in functional alterations in the bilateral V1 both in animals and humans [4]. Our results that the amblyopic patients had significantly thinner cortical thickness in bilateral V1 further demonstrated the abnormalities in V1 from the standpoint of structure. Meanwhile, the results were consistent with the previous studies using the similar analysis methods [9] or the VBM approaches [7, 8]. Compared with functional impairments, the structural deficits could directly reflect neuron loss in primary visual cortex. Moreover, we found that in the amblyopic patients, the cortical thicknesses of the bilateral V1 were negatively correlated with the amount of anisometropia, which might support the hypothesis that amblyopia arises from interocular suppressive interactions.

In addition, previous fMRI studies have found that visual dysfunctions occur beyond V1 in senior areas such as V2, V3, V4 and V5 or middle temporal complex (MT+) [14, 15]. Our results also found structural abnormalities in these visual areas, which provided anatomic evidence for senior visual dysfunction, such as color vision defects [16] and visuomotor deficits [17], in patients with amblyopia. It is noteworthy that in our results, the cortical thinning occurred unilaterally (primarily in the left hemisphere) in V2, V3, V4 and V5/MT+. Similarly, previous VBM studies also found structural abnormalities mainly in the left hemisphere [8, 18]. Since occipital lobe asymmetry has been reported in previous studies [19, 20], the more prominent anatomic differences on the left side between the amblyopic patients and controls could be due to differences in the maturation rate of the extrastriate cortex between the bilateral hemispheres. Moreover, the patients enrolled in this study were all right-handed, although they had amblyopia in either the left or the right eye. In the majority of right-handed humans, the left hemisphere is the language-dominant hemisphere. Previous studies have found that literacy will affect the early and senior visual areas, and the effects are mainly concentrated in the senior visual areas, especially for Chinese readers [21, 22]. Meanwhile there is quite little evidence that amblyopic reading is slower and more crowded [23, 24]. Therefore, the abnormal reading experiences might have resulted in the thinner cortical thicknesses of the left senior visual areas in the right-handed Chinese patients with amblyopia. These results suggest that laterality might exist in the development of senior areas of the visual cortex in patients with amblyopia.

In this study, we found that the mean curvature alterations were in the visual areas in which the cortical thickness was thinning, and the mean curvature of these visual areas seemed to increase in patients compared to controls. Curvature is a well-defined geometrical property that quantifies the nature and degree to which a surface deviates from being flat. Mean curvature can reflect folding of the small secondary and tertiary folds on the surface, and it has been used to study many neurodegenerative diseases [25, 26] to provide valuable insights into their multifactorial etiology. When cortical degeneration occurs in amblyopia, the imbalanced cortical thickness reduction in each visual area transformed the visual cortex from a flat into an uneven surface. The uneven surface gained spatial complexity and resulted in increased mean curvature. However, the mean curvature alterations showed statistical significance only in the bilateral V1. These results suggest that the curvature of the visual cortex may be another imaging marker that can indicate heterogeneous neural degeneration in patients with anisometropic amblyopia but not as precisely as cortical thickness. Moreover, no significant correlations were found between the mean curvature and any of the clinical variables in this study. It may indicate that the impact of clinical factors on the mean curvature was less obvious than that on the cortical thickness in patients with anisometropic amblyopia. In addition, as only three clinical variables were measured in this study, we might miss the potential clinical factors which could impact the mean curvature of the visual cortex. Regarding the surface area and cortical volume, we did not find a significant difference in these two surface parameters between the patients and controls in any visual areas.

The present study has several limitations. First, the sample size is quite small and only the older patients (older than age of 16) without effective treatment are recruited in the current study, which should make our results be regarded as preliminary. Larger populations and more clinical details, especially the treatment effect, are required to detect more accurate cortical reorganization in amblyopes. Second, only the first five (V1–V5) visual areas were analyzed in this study, and the visual areas were based on an anatomical atlas rather than retinotopic maps. Further studies should include more visual areas and use retinotopic approaches.

## Conclusion

Our results suggested that in addition to cortical thickness, the altered mean curvature of the cortex may indicate the neuroanatomic impairments of the visual cortex in patients with anisometropic amblyopia. Moreover, the structural changes presented bilaterally in the primary visual cortex, while the changes were unilateral in the secondary and more senior areas of visual cortex.

## Methods

### Participants

This study was approved by the Ethics Committee of Southwest Hospital. Written informed consent in accordance with the Declaration of Helsinki was obtained from all participants or their legal guardians. The method for participants recruitment has been described in our previous work [6]. Patients were recruited based on the criteria in the Expert Consensus on Amblyopia Diagnosis (2011) from the practices of collaborating ophthalmologists in Southwest Hospital, and normal-sight controls were recruited from the local community via advertisement. In total, 20 monocular anisometropic amblyopic patients and 20 normal-sight, age-, sex- and education-matched controls were enrolled in the study. All participants received comprehensive eye examinations that included assessments of visual acuity, cycloplegic refraction, intraocular pressure, simultaneous vision, fusion faculty, stereoscopic vision and slit lamp examination. Furthermore, all patients underwent optical coherence tomography (OCT) both for the amblyopic eyes and fellow eyes. Detailed information for the patients and controls is listed in Table 1. The patients mainly received occlusion therapy several years ago, but none of them obtained effective treatment. As the treatment outcomes were consistent in all patients, the treatment details were not described in the study. All participants were right-handed and had no history of strabismus, other ocular diseases, neurological disorders, or MRI contraindications.

### Data acquisition

MRI data were acquired at the Department of Radiology in Southwest Hospital via a 3.0 Tesla MR scanner (Trio Tim system; Siemens, Erlangen, Germany). A twelve-channel head coil was used as the radio frequency signal receiver. Tight but comfortable sponges were used to fix the head within the coil to minimize head motion, and a headset was used to reduce scanner noise. All participants were required to keep their eyes closed while remaining awake, and to keep their heads motionless during the scanning. Structural images were acquired using a magnetization-prepared rapid gradient echo imaging sequence with the following scan parameters: repetition time = 2530 ms, echo time = 2.34 ms, flip angle = 7°, matrix = 192 × 256, field of view = 256 × 256 mm^2^, slice thickness = 1 mm, and slice gap = 0.5 mm. A whole brain was composed of 192 high-resolution T1-weighted images in a sagittal view. The scan time was approximately 8 min.

### Data preprocessing

**Fig. 3:**
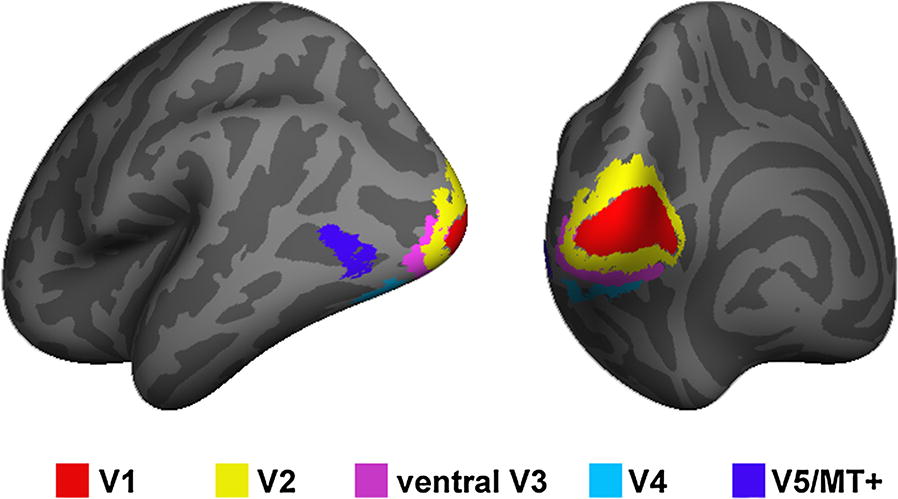
The V1, V2, ventral V3, V4 and V5/MT+ labels in the left hemisphere with a threshold at 80% probability

All preprocessing procedures for the MRI data were performed with our previously described methods [26, 27] via the FreeSurfer software package (version 5.3.0, http://surfer.nmr.mgh.harvard.edu). Cortical reconstruction and volumetric segmentation were automatically processed using the following procedures: removal of nonbrain tissue, Talairach transformation, segmentation of gray/white matter tissue, intensity normalization, and topological correction of the cortical surface and surface deformation to optimally place the tissue borders. After creating the cortical representations, the ROIs were parceled based on visual field maps V1–V5. The delineation of the V1 label was based on the study by Hinds et al. [28] and corresponded to Brodmann area (BA) 17, the V2 label was described by Fischl et al. [29] and corresponded to BA18, and the V5/MT+ label was based on the work of Malikovic et al. [30] and located close to the intersection of the anterior occipital and the inferior lateral occipital sulci in the region of the temporo-occipital junction. In addition, the ventral V3 and V4 labels were based on the Juelich histological atlas in FSL (http://fsl.fmrib.ox.ac.uk/fsl/fslwiki/Atlases/Juelich), as the V3 and V4 labels are not available in FreeSurfer. The FSL templates were transformed into surface labels and manually corrected according to the description by Rottschy [31]. The ventral V3 is buried deep in the collateral sulcus and the V4 is located on the lateral bank of this sulcus but also reaches the fusiform gyrus in the occipital section. The dorsal V3 was not delineated here because there are differing views regarding its anatomical location and additional subdivisions [12]. To avoid overlap among these labels, they were all thresholded at 80% probability (Fig. 3). Cortical thickness was calculated as the shortest distance between the gray matter (GM) and white matter (WM) surfaces at each vertex across the cortical mantle. The surface area was calculated as the area of the intermediate layer between the GM and WM surfaces. The gray matter volume at each vertex was determined by calculating the product of the surface area and the thickness at each surface vertex. The mean curvature was the average of the two principle curvatures (1/radius of an inscribed circle), and higher values represented a more steeply peaked curvature. Finally, we measured the average cortical thickness, surface area, cortical volume and mean curvature of all vertices in each ROI.

### Statistical analyses

Group differences in age and years of education between patients and controls were compared using independent two-sample t-tests, and sex differences were analyzed with Chi-square tests. Group differences in laterality of amblyopic/nondominant eye were compared using the Chi-square test. Interocular differences in RNFL thickness in patients were compared using paired sample t-test. As for other clinical variables, the cVA was converted to the logarithm of the minimum angle of resolution (LogMAR) values, the refractive power was expressed as spherical equivalent which was calculated as the sphere plus half of the cylinder, and the amount of anisometropia was quantified by the difference value of the binocular refractive power. Subsequently, Wilcoxon’s signed rank tests and Mann–Whitney U tests were performed to compare the paired and independent data, respectively. Differences in surface parameters (cortical thickness, surface area, cortical volume and mean curvature) between the patients and controls were compared using independent samples t-test with the following covariates: age, gender and gray matter volumes. Moreover, to determine whether the clinical variables correlated with any surface parameters, Pearson and Spearman rank correlation analyses between the clinical variables and the surface parameters were performed. The clinical variables included cVA of the amblyopic eyes, RNFL thickness, and the amount of anisometropia. All statistical analyses were performed using SPSS software (version 18.0; SPSS, Inc., Chicago, IL, USA) with a statistical significance setting at 0.05, and Bonferroni correction was used to determine the statistical significance level when multiple tests were performed in ROIs of the ipsilateral hemisphere.

## Data Availability

The datasets used and analyzed during the current study are available from the corresponding author on reasonable request.

## Abbreviations

fMRI: functional magnetic resonance imaging
SBM: surface-based morphometry
VBM: voxel-based morphometry
ROI: region of interest
cVA: corrected visual acuity
LogMAR: logarithm of the minimum angle of resolution
OCT: optical coherence tomography
RNFL: retinal nerve fiber layer
BA: Brodmann area
GM: gray matter
WM: white matter

## References

[1] Birch EE (2013). Amblyopia and binocular vision. Progr Retin Eye Res.

[2] Holmes JM, Clarke MP (2006). Amblyopia. Lancet.

[3] Repka MX, Kraker RT, Tamkins SM, Suh DW, Sala NA, Beck RW, Pediatric Eye Disease Investigator G (2009). Retinal nerve fiber layer thickness in amblyopic eyes. Am J Ophthalmol.

[4] Joly O, Franko E (2014). Neuroimaging of amblyopia and binocular vision: a review. Front Integr Neurosci.

[5] Ding K, Liu Y, Yan X, Lin X, Jiang T (2013). Altered functional connectivity of the primary visual cortex in subjects with amblyopia. Neural Plast.

[6] Liang M, Xie B, Yang H, Yu L, Yin X, Wei L, Wang J (2016). Distinct patterns of spontaneous brain activity between children and adults with anisometropic amblyopia: a resting-state fMRI study. Graefe’s Arch Clin Exp Ophthalmol.

[7] Mendola JD, Conner IP, Roy A, Chan ST, Schwartz TL, Odom JV, Kwong KK (2005). Voxel-based analysis of MRI detects abnormal visual cortex in children and adults with amblyopia. Hum Brain Mapp.

[8] Xiao JX, Xie S, Ye JT, Liu HH, Gan XL, Gong GL, Jiang XX (2007). Detection of abnormal visual cortex in children with amblyopia by voxel-based morphometry. Am J Ophthalmol.

[9] Du H, Xie B, Yu Q, Wang J (2009). Occipital lobe’s cortical thinning in ametropic amblyopia. Magn Reson Imaging.

[10] Hyde KL, Samson F, Evans AC, Mottron L (2010). Neuroanatomical differences in brain areas implicated in perceptual and other core features of autism revealed by cortical thickness analysis and voxel-based morphometry. Hum Brain Mapp.

[11] Fischl B (2012). FreeSurfer. NeuroImage.

[12] Wandell BA, Dumoulin SO, Brewer AA (2007). Visual field maps in human cortex. Neuron.

[13] Wiesel TN, Hubel DH (1963). Single-cell responses in striate cortex of kittens deprived of vision in one eye. J Neurophysiol.

[14] Conner IP, Odom JV, Schwartz TL, Mendola JD (2007). Monocular activation of V1 and V2 in amblyopic adults measured with functional magnetic resonance imaging. J AAPOS.

[15] Lerner Y, Hendler T, Malach R, Harel M, Leiba H, Stolovitch C, Pianka P (2006). Selective fovea-related deprived activation in retinotopic and high-order visual cortex of human amblyopes. NeuroImage.

[16] Kocak-Altintas AG, Satana B, Kocak I, Duman S (2000). Visual acuity and color vision deficiency in amblyopia. Eur J Ophthalmol.

[17] Grant S, Melmoth DR, Morgan MJ, Finlay AL (2007). Prehension deficits in amblyopia. Invest Ophthalmol Vis Sci.

[18] Li Q, Jiang Q, Guo M, Li Q, Cai C, Yin X (2013). Grey and white matter changes in children with monocular amblyopia: voxel-based morphometry and diffusion tensor imaging study. Br J Ophthalmol.

[19] Giedd JN, Snell JW, Lange N, Rajapakse JC, Casey BJ, Kozuch PL, Vaituzis AC, Vauss YC, Hamburger SD, Kaysen D (1996). Quantitative magnetic resonance imaging of human brain development: ages 4–18. Cereb Cortex.

[20] Maller JJ, Thomson RH, Rosenfeld JV, Anderson R, Daskalakis ZJ, Fitzgerald PB (2014). Occipital bending in depression. Brain.

[21] Szwed M, Qiao E, Jobert A, Dehaene S, Cohen L (2014). Effects of literacy in early visual and occipitotemporal areas of Chinese and French readers. J Cogn Neurosci.

[22] Strother L, Coros AM, Vilis T (2016). Visual cortical representation of whole words and hemifield-split word parts. J Cogn Neurosci.

[23] Levi DM, Song S, Pelli DG (2007). Amblyopic reading is crowded. J Vision.

[24] Kelly KR, Jost RM, De La Cruz A, Birch EE (2015). Amblyopic children read more slowly than controls under natural, binocular reading conditions. J AAPOS.

[25] Li S, Yuan X, Pu F, Li D, Fan Y, Wu L, Chao W, Chen N, He Y, Han Y (2014). Abnormal changes of multidimensional surface features using multivariate pattern classification in amnestic mild cognitive impairment patients. J Neurosci.

[26] Yu L, Yin X, Dai C, Liang M, Wei L, Li C, Zhang J, Xie B, Wang J (2014). Morphologic changes in the anterior and posterior subregions of V1 and V2 and the V5/MT+ in patients with primary open-angle glaucoma. Brain Res.

[27] Yu L, Xie L, Dai C, Xie B, Liang M, Zhao L, Yin X, Wang J (2015). Progressive thinning of visual cortex in primary open-angle glaucoma of varying severity. PLoS ONE.

[28] Hinds OP, Rajendran N, Polimeni JR, Augustinack JC, Wiggins G, Wald LL, Diana Rosas H, Potthast A, Schwartz EL, Fischl B (2008). Accurate prediction of V1 location from cortical folds in a surface coordinate system. NeuroImage.

[29] Fischl B, Rajendran N, Busa E, Augustinack J, Hinds O, Yeo BT, Mohlberg H, Amunts K, Zilles K (2008). Cortical folding patterns and predicting cytoarchitecture. Cereb Cortex.

[30] Malikovic A, Amunts K, Schleicher A, Mohlberg H, Eickhoff SB, Wilms M, Palomero-Gallagher N, Armstrong E, Zilles K (2007). Cytoarchitectonic analysis of the human extrastriate cortex in the region of V5/MT+: a probabilistic, stereotaxic map of area hOc5. Cereb Cortex.

[31] Rottschy C, Eickhoff SB, Schleicher A, Mohlberg H, Kujovic M, Zilles K, Amunts K (2007). Ventral visual cortex in humans: cytoarchitectonic mapping of two extrastriate areas. Hum Brain Mapp.

